# Mid-term morphological changes in Frozenix

**DOI:** 10.1093/icvts/ivaf104

**Published:** 2025-05-08

**Authors:** Takanobu Kimura, Hiroshi Tsuneyoshi, Shuji Setozaki, Hideyuki Katayama, Akira Takeuchi, Takeru Nakamura, Koki Tamaoka

**Affiliations:** Department of Cardiovascular Surgery, Shizuoka Prefecture General Hospital, Shizuoka, Shizuoka, Japan; Department of Cardiovascular Surgery, Shizuoka Prefecture General Hospital, Shizuoka, Shizuoka, Japan; Department of Cardiovascular Surgery, Shizuoka Prefecture General Hospital, Shizuoka, Shizuoka, Japan; Department of Cardiovascular Surgery, Shizuoka Prefecture General Hospital, Shizuoka, Shizuoka, Japan; Department of Cardiovascular Surgery, Shizuoka Prefecture General Hospital, Shizuoka, Shizuoka, Japan; Department of Cardiovascular Surgery, Shizuoka Prefecture General Hospital, Shizuoka, Shizuoka, Japan; Department of Cardiovascular Surgery, Shizuoka Prefecture General Hospital, Shizuoka, Shizuoka, Japan

**Keywords:** frozen elephant trunk, aortic surgery, aortic aneurysms, aortic dissection, morphological change

## Abstract

**OBJECTIVES:**

The frozen elephant trunk is widely used for aortic arch aneurysms and DeBakey type I and III aortic dissections. However, radial force and spring-back of the stent can cause morphological changes, contributing to stent-induced new entry. This study aimed to evaluate postoperative morphological changes of frozen elephant trunk and provide biomechanical insights.

**METHODS:**

This retrospective study included 107 patients who underwent Frozenix placement between April 2014 and December 2023. Clinical outcomes and morphological changes of frozen elephant trunk were assessed. Morphological changes were evaluated by measuring the distal diameter and stent angle, defined as the angle between the lines perpendicular to the proximal and distal ends of the stent, via computed tomography, including angiographic imaging.

**RESULTS:**

Thirty-day mortality was 3%. The median follow-up duration was 24 months, and the incidence of all-cause mortality was 9.69 events per 100 patient years. Stent-induced new entry occurred in seven patients (7%). The distal diameter was enlarged in all 95 evaluated patients; 59 (62%) exceeded the stent size. In these patients, the mean distal diameter reached 105% of the original stent size. Dissections demonstrated greater distal diameter expansion than thoracic aortic aneurysms. The stent angle, assessed in 33 patients, increased in all cases, with progressive changes observed over 3 years.

**CONCLUSIONS:**

Postoperative enlargement of the distal diameter beyond the original stent size and progressive changes in stent angle should be taken into account when selecting the size of the frozen elephant trunk and planning the surgical strategy. Long-term computed tomography follow-up is also warranted.

## Graphical Abstract

**Figure:**
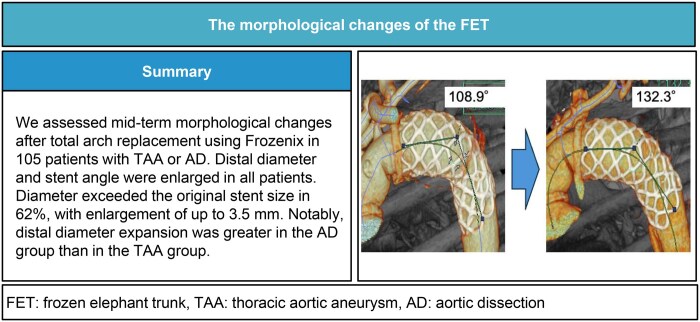

## INTRODUCTION

In 1996, Kato *et al.* [1] reported on the first surgery to use a custom-made device that combined Dacron and stent grafts in thoracic aortic surgery. In 2003, Karck *et al.* [2] used the Chavan–Haverich device (Curative Medical Devices GmbH, Dresden, Germany) and named it ‘frozen elephant trunk (FET)’. FET is used to treat extensive aortic dissections and thoracic aortic aneurysms involving the distal arch and more distal segments, primarily to promote favourable aortic remodelling in these regions, and different commercial devices have been introduced globally. Frozenix (Japan Lifeline, Tokyo, Japan) was approved in Japan for insurance reimbursement in 2014 [3], making FET a standard procedure for thoracic aortic surgery in Japan. The FET is known for its high aortic remodelling effect and ability to perform distal anastomosis at a shallow position during aortic surgery [3–5]. However, stent-induced new entry (SINE) has been reported in approximately 15–18% of cases, which is a relatively high incidence [6], although most SINE cases are asymptomatic. SINE causes rapid aortic expansion or rupture, which has a mortality rate of 28% [7–10]. The implicated risk factors for SINE include chronic aortic dissection, oversizing, stent length and forces inherent to the FET, such as the radial force, which expands the stent outwardly in a radial direction, and the spring-back force, a characteristic of nitinol that enables the stent to return to its original shape [6, 11, 12]. Few studies have investigated the duration of these forces and their effect on the stent’s shape over time [13, 14]. Elucidating the long-term morphological changes of FET is crucial to improve follow-up strategies. Therefore, we conducted a single-centre retrospective study to investigate the morphological changes in FET during mid-term follow-up in patients who underwent Frozenix placement for distal aortic arch aneurysms or DeBakey type I or III aortic dissections between April 2014 and December 2023. The primary end-point of this study was the morphological change of FET, whereas secondary end-points included postoperative mortality, other clinical outcomes, aortic remodelling and aortic reinterventions.

## PATIENTS AND METHODS

### Ethical statement

This single-centre, retrospective observational study obtained approval from the Ethics Committee of Shizuoka Prefecture General Hospital on 6 August 2024 (protocol number SGHIRB#2024023). This study adhered to ethical standards under the 1964 Helsinki Declaration and its amendments. It was conducted in accordance with the STROBE criteria for retrospective studies. Written informed consent was waived due to the retrospective nature of the study. The study data were collected and stored in accordance with the ethical guidelines outlined in the WMA Declaration of Taipei. The establishment and ongoing management of the study database were reviewed and approved by the Ethics Committee of Shizuoka Prefecture General Hospital.

### Patient characteristics

The FET was used in patients with distal aortic arch aneurysms or DeBakey type I or III aortic dissections involving extensive aortic disease. Patient selection flowchart is presented in Supplementary Fig. S1. This retrospective observational study included patients who underwent Frozenix placement at our hospital between April 2014 and December 2023. Clinical outcomes were analysed in all patients, whereas morphological changes were assessed in patients who underwent non-contrast or contrast-enhanced computed tomography (CT), including CT angiography (CTA), with a minimum follow-up period of 1 month. The present study aimed to evaluate morphological changes in the FET; therefore, patients with a history of thoracic endovascular aortic repair (TEVAR) or descending aortic replacement were excluded. Patients who were treated with the original Frozenix, introduced in 2014, were included, whereas those treated with the newer Frozenix Partial ET, launched in 2023, were excluded. Frozenix was the only FET device available in our institution during the study period. During the study period, no patient underwent aortic surgery using Thoraflex Hybrid (Vascutek, Inchinnan, UK), which was also approved for insurance in Japan in July 2023. Additionally, E-vita Open NEO (JOTEC, Hechingen, Germany) has not been approved for insurance coverage in Japan.

Aortic dissection occurring more than 3 months after onset were defined as chronic, whereas those occurring between 2 weeks and 3 months after onset were defined as acute.

### Operative procedures

Preoperatively, we used a three-dimensional (3D) imaging analysis system to determine the circumference of the entire aorta and the true lumen at the intended placement site, calculating the vessel diameter in perpendicular view. We selected stents that were 105–120% of the distal vessel’s diameter for true aneurysms and 105–110% of the true lumen’s diameter for aortic dissection cases, following the manufacturer’s recommendations. In cases where 3D image analyses were unavailable, the FET size was defined as 105% of the maximum diameter of the true lumen. The FET’s length was selected to position its distal end in the straight segment of the descending aorta, avoiding extension beyond the level of the seventh thoracic vertebrae to minimize the risk of spinal cord injury (SCI).

In cases of aortic arch aneurysms, cardiopulmonary bypass (CPB) was established via the ascending aorta with single venous drainage as the standard strategy. For aortic dissection cases, CPB was established via the femoral and axillary arteries, with double venous drainage through the superior and inferior vena cava as the basic approach. Antegrade cerebral perfusion under circulatory arrest and moderate hypothermia (25°C–28°C) were used for the surgical process. The FET graft was transected, leaving 5–10 mm of the graft from the stent. Distal anastomosis was established distal to the left subclavian artery or between the left common carotid and the left subclavian arteries. When performing the distal anastomosis between the left common carotid artery and the left subclavian artery, the left subclavian artery reconstruction was achieved using one of the following two methods. In cases where the fenestration technique was used, following the report by Okamura *et al.* [15], a fenestration was created through an ∼10-mm hole in the FET graft at the origin of the left subclavian artery using scissors. The other method involved reconstructing the left subclavian artery by anastomosing an 8-mm graft to the exposed axillary artery below the clavicle, guiding the graft through the thoracic cavity, and connecting it to a 4-branched graft.

### Clinical outcomes

Postoperative follow-up was conducted at 1 month, 6 months and 1 year after surgery, and annually thereafter. During the follow-up period, the survival status and cause of death were investigated through direct outpatient visits and telephone inquiries. The follow-up period was concluded on 28 February 2024.

Patients with neurological deficits underwent brain MRI. Those with acute-phase cerebral infarction findings were classified as having cerebral infarction, and transient ischaemic attacks were excluded. SCI was defined as acute-phase infarction identified on spinal MRI or lower limb paralysis and bladder or rectal dysfunction, where cerebral infarction was ruled out.

### CTA data acquisition and image reconstruction methods

**Figure 1:**
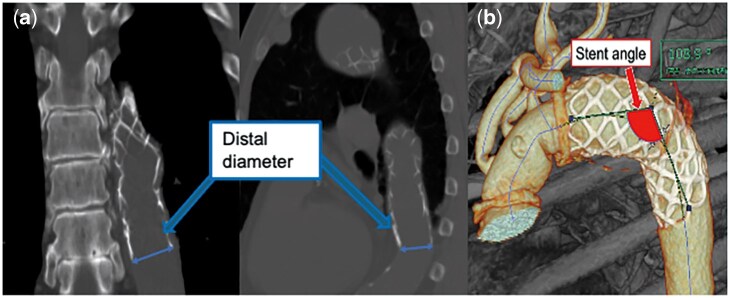
Measurement of stent angle and distal diameter. (**a**) Method for measuring the stent angle, i.e. the angle formed between the proximal and distal ends of the stent. (**b**) Method for measuring the distal diameter of the stent. The distal diameter is the diameter at the distal point of the stent and is calculated as the average of measurements obtained from computed tomography coronal and sagittal views.

We retrospectively investigated how the distal diameter and stent angle of Frozenix changed. The distal diameter was measured in the coronal and sagittal sections, and their average was considered as the distal diameter. Changes in distal diameter were analysed using follow-up data that were available for up to 5 years. Consistent with a previous report, we defined the stent angle as the angle between the proximal and distal ends of the stent graft [11] and measured it using the 3D imaging analysis system, Vincent (Fujifilm Corporation, Tokyo, Japan) (Fig. 1a and b). CT follow-ups were performed at discharge, 1 month, 6 months postoperatively, and annually thereafter. Cases that required additional treatments for the distal aorta post-FET, such as TEVAR or descending aortic replacement, were excluded from the follow-up analysis of morphological changes after the additional intervention, as these treatments could alter the natural course of the morphological changes in the FET.

### Statistical analysis

Continuous variables were assessed for normality using the Shapiro–Wilk test. Normally distributed data were presented as mean (standard deviation [SD]), and non-normally distributed data as medians with interquartile range (IQR). Categorical variables were expressed as counts and percentages. Logistic regression was used to analyse the association between FET length and SCI incidence. Kaplan–Meier curves estimated all-cause mortality, and the log-rank test compared groups. A mixed-effects linear regression model assessed the effect of time on the stent angle. All analyses were performed using SPSS (version 30, IBM Corp., Armonk, NY, USA). A *P*-value <0.05 was considered statistically significant.

## RESULTS

### Patient characteristics

Table 1 presents the patient characteristics. Among the 109 patients who underwent Frozenix placement at our hospital during the study period, patient characteristics, surgical data and clinical outcomes were analysed for 107 patients, excluding two cases that used partial ET. Seven patients (6%) had a history of cardiac surgery for ascending aortic replacement caused by Stanford type A acute aortic dissection, followed by redo surgery using FET implantation due to distal arch expansion.

**Table 1:** Patient characteristics

Variables	*n* = 107
Age (IQR)	75.0 (68.5–79.0)
Male, *n* (%)	90 (84)
Hypertension, *n* (%)	97 (91)
Hyperlipidaemia, *n* (%)	45 (42)
Diabetes, *n* (%)	13 (12)
Chronic kidney disease, *n* (%)	66 (62)
Haemodialysis, *n* (%)	5 (5)
Previous cardiac surgery, *n* (%)	7 (7)
Stroke, *n* (%)	9 (8)
COPD, *n* (%)	9 (8)
Indication for operation, *n* (%)	
Acute aortic dissection	29 (27)
Chronic aortic dissection	16 (15)
Aortic aneurysm	62 (58)
Extent of aneurysms in TAA 62 cases, *n* (%)	
Zone 3	42/62 (68)
Zone 4	17/62 (27)
Extended aneurysms^a^	3/62 (5)
Stanford classification in AD 45 cases, *n* (%)	
Type A	32/45 (71)
Type B	13/45 (29)
Extent of dissection in AD 45 cases, *n* (%)	
Zone 4	4/45 (9)
Zone 4	41/45 (91)
Location of the entry in AD 45 cases *n* (%)	
Ascending aorta	8/45 (18)
Aortic arch	12/45 (27)
Descending aorta	19/45 (42)
Unknown	6/45 (13)

Data are expressed as frequencies (%) or medians (IQR).

a Aortic aneurysms that extend continuously from the descending thoracic aorta to beyond the thoraco-abdominal aorta.

AD: aortic dissection; COPD: chronic obstructive pulmonary disease; IQR: interquartile range; TAA: thoracic aortic aneurysm.

### Operative data and postoperative outcomes

Table 2 summarizes the surgical data. Although various lengths of FET were used, longer prostheses were used in cases involving extensive aortic aneurysms or when proximal anastomosis was performed in zone 2 instead of zone 3. Table 3 depicts the postoperative outcomes. Median follow-up period was 24 (10.0–48.0) months. Five (5%) patients experienced SCI postoperatively, two and three of which were transient and permanent, respectively. The logistic regression evaluating the association between the FET length and SCI incidence revealed an odds ratio of 1.08 (95% confidence interval 0.76–1.54) for SCI occurrence per 1-mm increase in FET length, indicating no statistically significant association (*P* = 0.656). A total of 18 patients (17%) developed cerebral infarction. However, six of these cases underwent emergency surgery without preoperative brain MRI, including three cases with coma. SINE was observed in seven cases (7%), including two of true aneurysm and five of aortic dissection. All cases demonstrated no aortic diameter expansion with conservative treatment; thus, no reoperations were required. Aortic reintervention was performed in 16 cases (15%), including 15 TEVAR cases (8 for false lumen expansion, 5 for aneurysm expansion, 1 for re-dissection and 1 for aortic rupture), and 1 descending aorta replacement for a descending aortic aneurysm. The incidence of reintervention was 6.92 events/100 patient years, and the median time to reintervention was 14.5 (IQR 6.0–38.0) months. No additional treatment was required for FET-related complications. Supplementary Fig. S2 illustrates the changes in the aortic diameter at the distal end of FET. Reintervention was performed in 12% (13 cases) of cases due to expansion of the false lumen or aneurysm in the distal portion of the FET; however, most cases exhibited no significant changes or demonstrated a reduction in aortic diameter.

**Table 2:** Surgical data

Variables	*n* = 107
Emergency or urgent, *n* (%)	29 (27)
Concomitant surgery, *n* (%)	33 (31)
CABG	18 (17)
AVR	8 (8)
PVI or MAZE or/and LAAO	9 (8)
Others	2 (2)
Operation time, min (IQR)	393.0 (350.5–466.0)
Aorta clamp time, min (IQR)	134.0 (111.0–155.5)
Cardio–pulmonary bypass time, min (IQR)	207.0 (187.0–248.0)
Arrest of lower body time, min (IQR)	51.5 (44.0–57.0)
Bleeding, ml (IQR)	1454 (897.5–2423.2)
Distal anastomosis zone, *n* (%)	
Zone 2	63 (59)
Zone 3	44 (41)
Length of FET, *n* (%)	
6 cm	15 (14)
9 cm	25 (23)
12 cm	52 (49)
15 cm	15 (14)
Diameter of FET, mm (IQR)	31.0 (27.0–33.0)

Data are expressed as frequencies (%) or medians (IQR).

AVR: aortic valve replacement; CABG: coronary artery bypass grafting; FET: frozen elephant trunk; IQR: interquartile range; LAAO: left atrial appendage occlusion; PVI: pulmonary vein isolation.

**Table 3:** Postoperative outcomes

Variables	*n* = 107
Hospital day	19.0 (13.0–30.0)
Stent-induced new entry, *n* (%)	7 (7)
Stroke, *n* (%)	18 (17)
Spinal cord injury, *n* (%)	5 (5)
Acute kidney injury, *n* (%)	5 (5)
Reintervention for aorta, *n* (%)	16 (15)
30-days mortality, *n* (%)	3 (3)
Aorta related death, *n* (%)	1 (1)
Follow-up duration	24.0 (10.0–48.0)

Data are expressed as frequencies (%) or medians (IQR).

**Figure 2:**
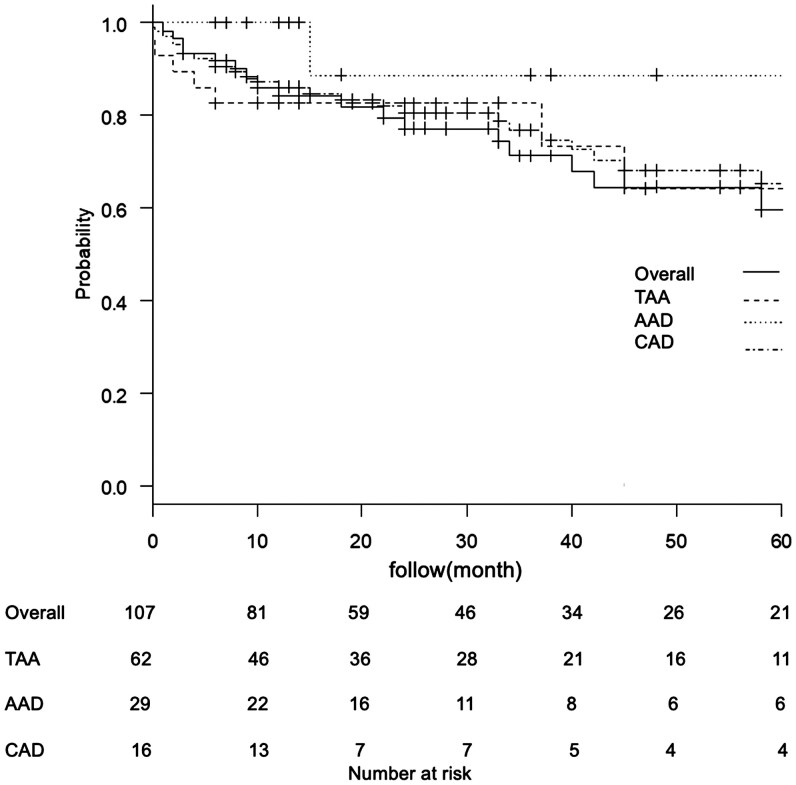
Kaplan–Meier survival curves for overall survival. The Kaplan–Meier curves of the entire cohort as well as the subgroups of patients with thoracic aortic aneurysm (TAA), acute aortic dissection (AAD) and chronic aortic dissection (CAD). No significant differences in overall mortality were observed among the disease groups.

The 30-day mortality rate was 3% (three cases), including two cases of cerebral infarction and one case of nonocclusive mesenteric ischaemia. Figure 2 shows the Kaplan–Meier curves for overall mortality of all patients and for patients categorized according to disease. The log-rank analysis revealed no significant difference in the mortality rates among the disease groups (*P* = 0.892). The incidence of all-cause mortality was 9.69 events/100 patient years; however, aortic-related mortality was limited to one case, which involved rupture of an aortic root aneurysm following surgery for Stanford type A acute aortic dissection. Most other deaths were caused by nonsurgical, late-phase complications, such as infections, cerebrovascular complications or cancer. The overall mortality for hemiarch replacement performed in the same period at our institution was 9.34 events/100 patient years, showing no significant difference compared to FET. Clinical outcomes based on disease are presented in Supplementary Table S1.

### Morphological change of FET

**Figure 3:**
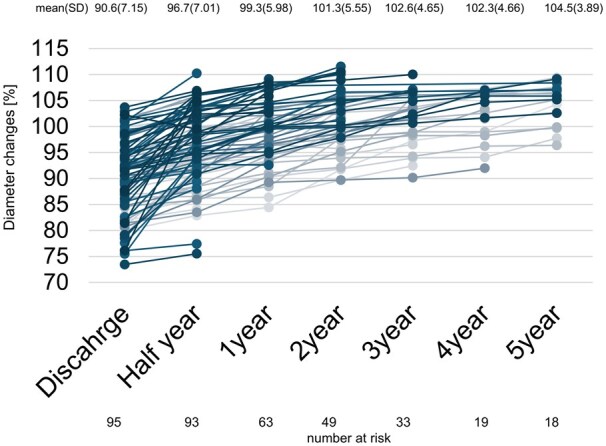
Changes in distal diameter over time. This line graph illustrates the changes in distal diameter over time. The vertical axis represents the percentage relative to the original product size (100%), and the horizontal axis indicates the time in years.

Evaluation of the distal aortic diameter was performed in 95 patients who were followed with CTA or CT for more than 1 month. The remaining 12 patients, including 3 patients who were lost to follow-up during the first postoperative month, 2 patients who underwent TEVAR during the first postoperative month, 4 patients who underwent FET after prior TEVAR and 3 patients who died in the perioperative period, were excluded from the morphological analysis (Fig. 1). Figure 3 illustrates the changes in distal diameter. The vertical axis represents the percentage of distal diameter relative to the product size (100%). All 95 patients demonstrated an increase in distal diameter from the time of placement to the mid-term follow-up. The mean distal diameter increased from 90.6 ± 7.15% at discharge to 99.3 ± 5.98% at 1 year, and reached 104.5 ± 3.89% at 5 years. As the distal diameter continued to expand, it converged towards the product size, and 59 (62%) cases had already exceeded the product size. Comparison of changes in distal diameter by disease revealed that thoracic aortic aneurysms showed a relatively gradual expansion, whereas acute and chronic aortic dissections demonstrated significant changes in diameter from discharge to 6 months post-discharge, with continued expansion afterward (Supplementary Fig. S3a–c).

**Figure 4:**
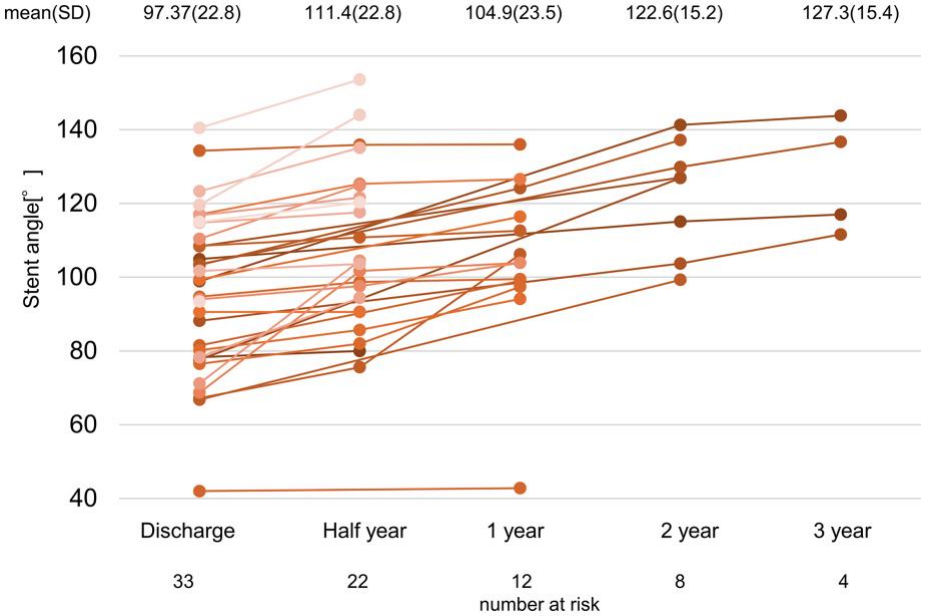
Changes in stent angle. The line graph illustrates the changes in stent angle from the immediate postoperative period until 3 years after the surgery. The vertical axis represents the stent angle, and the horizontal axis represents time in years.

Figure 4 illustrates the changes in the stent angle. Out of the 107 patients, stent angle measurements could only be performed in 33 patients using 3D imaging analysis system, with the follow-up period extending up to 3 years. All cases demonstrated stent angle expansion, with a median angle change of 5.5° (IQR: 2.25–13.1) in the first 6 months. The mixed-effects linear regression model to evaluate the effect of time on the stent angle revealed that time had a significant effect on the stent angle (*F*(1, 72.865) = 1618.047, *P* < 0.001), indicating that the stent angle significantly changed over time. In contrast, the disease type did not have a significant effect on the stent angle (*P* = 0.499). Supplementary Fig. S4 illustrates a typical case of chronic aortic dissection. The stent angle increased by ∼3° at 6 months postoperatively, with a significant change of 20° at 1 year. The expansion continued even at 2 years postoperatively.

## DISCUSSION

The major findings of this study are as follows: (i) morphology of the FET, assessed based on changes in the distal stent diameter and the stent angle, continued to change up to the mid-term period, and (ii) 62% of the patients exhibited expansion beyond the labelled size of the FET. These findings provide new insights into the long-term morphological changes of FET, which have been scarcely reported, and offer new perspectives on FET size selection.

The FET offers several advantages, including high aortic remodelling effect and distal anastomosis at a shallow level during arch replacement. In Japan, several studies have reported favourable outcomes using Frozenix [3–5, 13]. Similarly, favourable remodelling outcomes have also been reported for other FET devices, such as Thoraflex Hybrid Prosthesis and E-vita Open [16].

Current knowledge about the radial and spring-back forces of FET remains limited. Factors that regulate these forces include the material, design and length of the stent. Most FET devices, including Frozenix, are made of Nitinol, which exhibits the shape-memory characteristics that generate the radial and spring-back forces. These properties may contribute to long-term morphological changes of FET. The diameter of the wire of the newer Frozenix Partial ET is ∼10% smaller than that of the original Frozenix, resulting in a 43% reduction in the spring-back force and a 29% reduction in the radial force. This highlights that wire thickness is also a crucial factor in regulating stent forces.

Kreibich *et al.* conducted an *ex vivo* experiment on the association between stent structure and stiffness, comparing the radial force of Thoraflex and E-vita by applying mechanical pressure to the distal ends of the stents. Thoraflex exhibited greater stiffness, which was related to its ring-type structure at the distal end and might be associated with its higher rate of SINE [6]. No similar studies have focused on Frozenix; however, Frozenix exhibits a distinct structure compared to Thoraflex and E-vita, indicating that radial and spring-back forces may vary. Thoraflex and E-vita comprise independent wires, whereas Frozenix is a woven-type stent formed by a single wire. Woven stents are considered to demonstrate a stronger spring-back force than independent stents [3, 8]. In the present study, the stent angle changed by 5.5° over 6 months, exceeding the 4° expansion over 3.8 years reported by Osswald *et al.* [12] for E-vita. However, Frozenix has an internal skeleton; thus, the direct stress on the aorta may be less than the external skeleton stents.

Remarkably, in this patient group, expansion beyond the product size of the FET was observed in 62% of cases. Similarly, Hayashi *et al.* [14] analysed FET morphology in 45 cases using Frozenix and reported that it expanded beyond its product size in 95.6% of cases. Frozenix uses a woven graft, which is known to expand after placement under the influence of blood flow [17]. Additionally, the stent used in Frozenix is ∼10% larger than the size of the graft itself; for example, a 27-mm FET contains a stent ∼30 mm in size. Therefore, it is reasonable to expect that expansion beyond the product size occurs after the implantation of Frozenix. If there is concern about sizing when selecting the FET, choosing a smaller size might be advisable, considering potential oversizing.

Although the all-cause mortality rate was relatively high (28 cases, 9.69 events/100 patient years) during the follow-up period, aortic-related death occurred in only one patient. The high overall mortality rate was influenced by the inclusion of older adults and high-risk patients with multiple comorbidities. Moreover, most deaths were unrelated to the surgery. In a study of 121 cases of FET using Frozenix with long-term follow-up, Tokunaga *et al.* [18] reported an aortic-related mortality rate of 3% (four cases), including aneurysmal rupture, graft infection and non-occlusive mescentric ischemia. These results are comparable to our findings, suggesting the high potential of FET in reducing aortic-related mortality. As previously reported by some studies [19–21], FET is a safe procedure even in resternotomy cases, such as those with a history of ascending aorta replacement for Stanford type A aortic dissection followed by enlargement of the aortic arch. In this study, seven patients (6%) underwent resternotomy, and their outcomes were comparable to those of primary surgery cases.

A total of 18 patients (16.8%) developed cerebral infarction. Among these, six underwent emergency surgery without preoperative brain MRI, including three patients with coma, suggesting the possibility of pre-existing cerebral infarction. Berger *et al.* [16] reported cerebral infarction in 13% of patients undergoing FET for aortic dissection using the Thoraflex and E-vita Open, whereas Ogino *et al.* [3] reported a cerebral infarction rate of 10% in patients undergoing FET with Frozenix. Based on these findings, we believe that there is no significant difference in the incidence of cerebral infarction among different devices.

Kreibich *et al.* [6] reported a d-SINE incidence of 13% with Thoraflex and E-vita, which was comparable to that reported for Frozenix, estimated at ∼14% [7, 11], though some studies have reported higher incidences [22]. Given that our study had a relatively short follow-up period, the incidence of SINE may have been underestimated. The causes of SINE include radial and spring-back forces, oversizing and longitudinal strain mismatch [7, 11]. Hiraoka *et al.* [22] reported that the cumulative incidence of SINE after FET placement increased over several years after the procedure, which may be related to the persistent radial and spring-back forces. Symptomatic d-SINE accounts for ∼5% of all SINE cases. However, once SINE occurs, it can lead to rapid aortic enlargement or rupture, with an extremely high mortality rate of 25% [7–10]. Thus, postoperative surveillance of morphological changes of FET and the occurrence of SINE is crucial, and regular CT follow-up is warranted.

### Limitations

This study has several limitations. First, it is a small-scale retrospective study with limited number of cases, preventing comprehensive statistical analysis. Especially, the analysis of stent angle was performed in only 33 cases, which is an extremely small sample size, limiting the generalizability of the findings. Second, the observation period is short, and further long-term studies on the morphological changes of Frozenix are warranted. Another limitation of this study is the lack of a comparative group, such as patients treated with TEVAR. In addition, the study population included heterogeneous diagnoses, such as aneurysms, acute and chronic dissections, and redo cases, which may have influenced the morphological outcomes.

## CONCLUSION

The sustained expansion of the distal diameter and stent angle observed in this study suggests that the radial and spring-back forces of FET persist through the mid-term postoperative period. These findings emphasize the importance of careful follow-up after FET placement to monitor ongoing morphological changes and the potential risk of SINE.

## Supplementary Material

ivaf104_Supplementary_Data

## Data Availability

The data underlying this article are available in the article and its online supplementary material.

## ABBREVIATIONS

CT: Computed tomography
CTA: Computed tomography angiography
FET: Frozen elephant trunk
MRI: Magnetic resonance imaging
SCI: Spinal cord injury
SINE: Stent-induced new entry
TEVAR: Thoracic endovascular aortic repair
