# Supplementary material for: Mid-term morphological changes in Frozenix
Source: Interdiscip Cardiovasc Thorac Surg. 2025 May 8;40(5):ivaf104. doi: 10.1093/icvts/ivaf104 (PMC12101869; doi:10.1093/icvts/ivaf104)
Supplement: ivaf104_Supplementary_Data [file ivaf104_supplementary_data.zip › Supplementary Figures legends S1 to S4 3rd version.docx]

**Supplemental figures**

Supplementary Figure S1. Flowchart of the patient selection criteria
FET, frozen elephant trunk; TEVAR, thoracic endovascular aortic repair; CT, computed tomography

Supplementary Figure S2. Changes in distal aortic diameter after FET placement

This figure illustrates the progression of distal aortic diameter at the FET landing zone, presented for the entire cohort and stratified by disease group. While some cases showed an increase in aortic diameter, most demonstrated favorable aortic remodeling. (a) Overall, (b) thoracic aortic aneurysm, (c) acute aortic dissection, (d) chronic aortic dissection

Supplementary Figure S3. Changes in distal diameter of FET by disease type.

(a) Thoracic aortic aneurysm, (b) acute aortic dissection, (c) chronic aortic dissection

Supplementary Figure S4. Representative case of stent angle changes

Expansion of the stent angle began 6 months postoperatively, with significant changes occurring within the first year. The stent angle continued to increase even beyond 2 years postoperatively.
