# Supplementary material for: Mid-term morphological changes in Frozenix
Source: Interdiscip Cardiovasc Thorac Surg. 2025 May 8;40(5):ivaf104. doi: 10.1093/icvts/ivaf104 (PMC12101869; doi:10.1093/icvts/ivaf104)

**Supplemental Tables**

Supplementary Table S1. Clinical outcomes categorized by disease

| Disease variables | Aortic aneurysms  (n = 62) | Acute aortic dissection  (n = 29) | Chronic aortic dissection  (n = 16) |
| --- | --- | --- | --- |
| Hospital days (IQR) | 18.0 (13.0–31.0) | 27.0 (17.0–33.0) | 14.5 (11.0–21.5) |
| Stent-induced new entry, n (%) | 2 (3) | 2 (7) | 0 (0) |
| Stroke, n (%) | 10 (16) | 7 (24) | 1 (6) |
| Spinal cord injury, n (%) | 4 (6) | 1 (3) | 0 (0) |
| Acute kidney injury, n (%) | 3 (4) | 2 (7) | 0 (0) |
| Reintervention for the aorta, n (%) | 7 (11) | 7 (24) | 3 (19) |
| 30-day mortality, n (%) | 1 (2) | 2 (7) | 0 (0) |
| Follow up period, months (IQR) | 25.0 (9.2–52.5) | 24.0 (10.0–45.0) | 26.0 (11.5–48.5) |

Data are expressed as n (%) or medians (IQR).


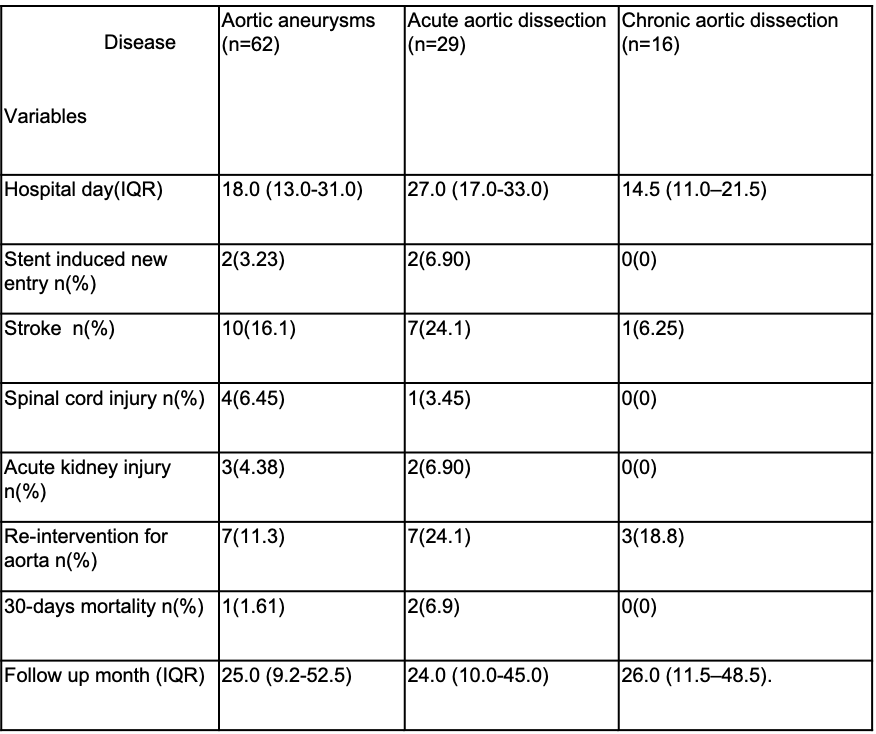

Supplement: ivaf104_Supplementary_Data [file ivaf104_supplementary_data.zip › Supplementary Tables legends S1 3rd version.docx]
