# Supplementary figures and images for: Mid-term morphological changes in Frozenix
Source: Interdiscip Cardiovasc Thorac Surg. 2025 May 8;40(5):ivaf104. doi: 10.1093/icvts/ivaf104 (PMC12101869; doi:10.1093/icvts/ivaf104)

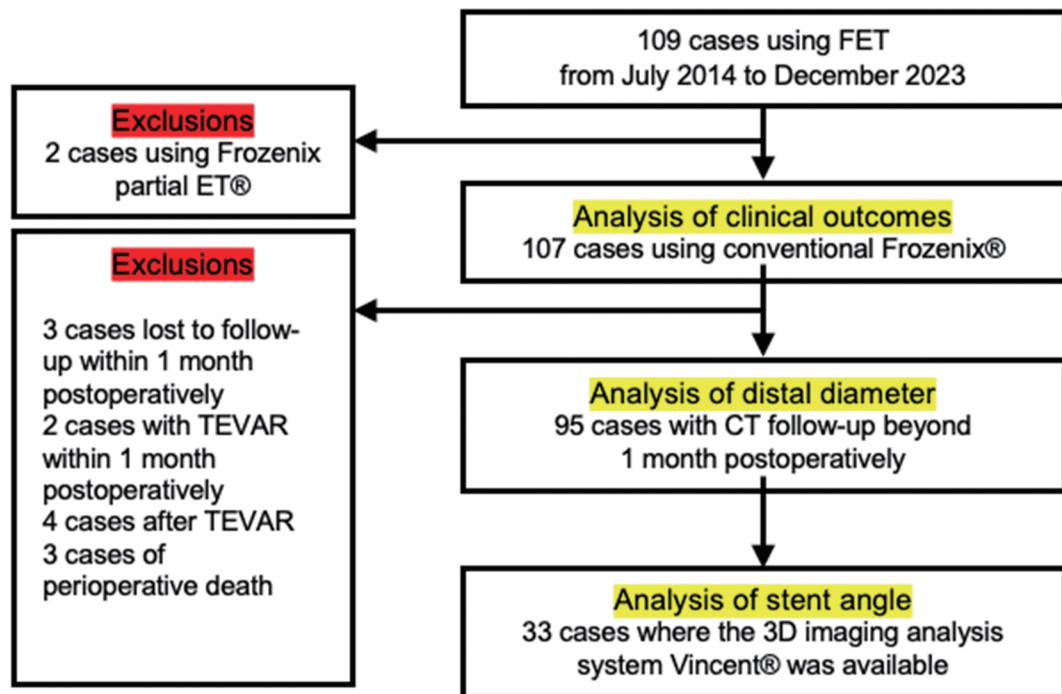

mean(SD) 35.1(5.15) 35.2(5.40) 35.2(4.85) 35.5(5.00) 36.0(4.90) 37.2(4.86) 37.6(4.39) 38.4(4.96)

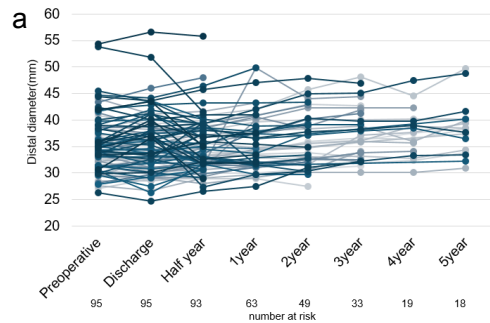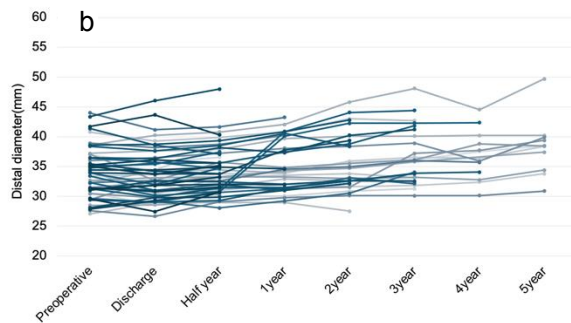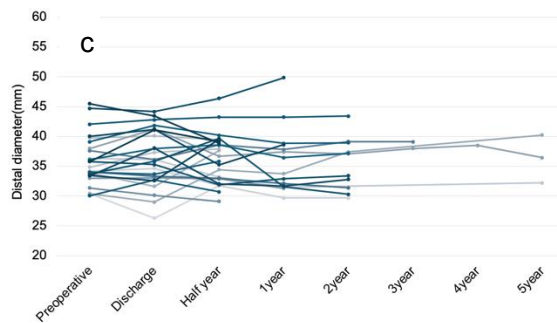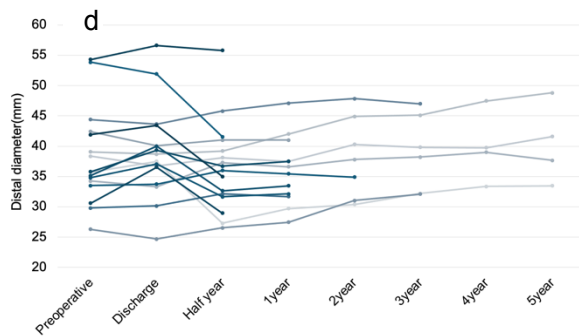

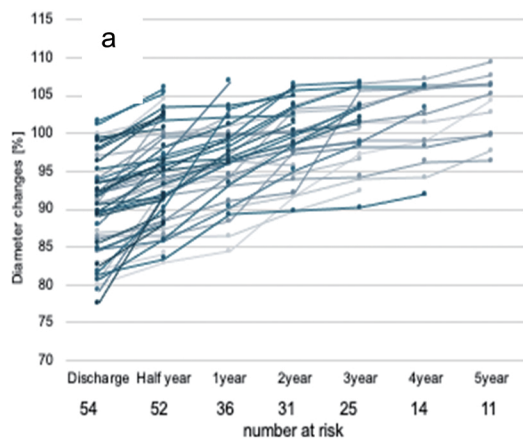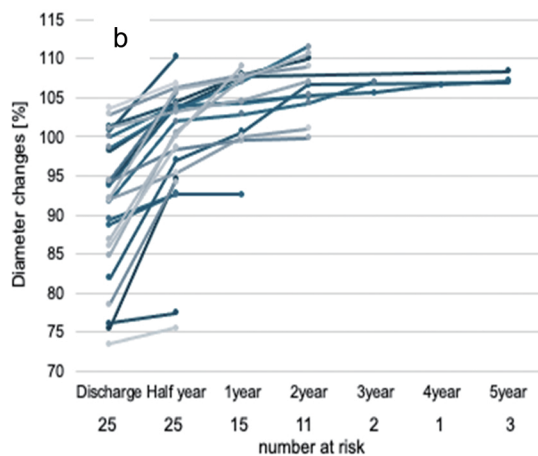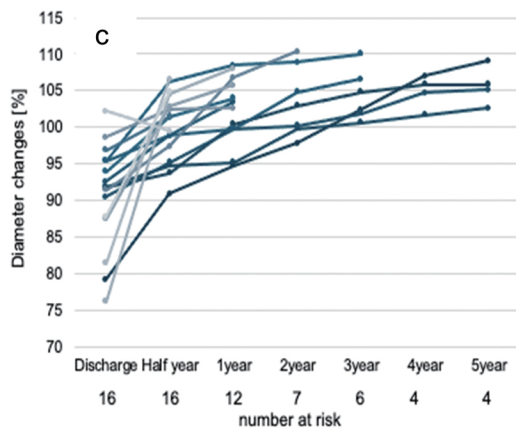

Supplement: ivaf104_Supplementary_Data [file ivaf104_supplementary_data.zip › Supplemental figure 1]

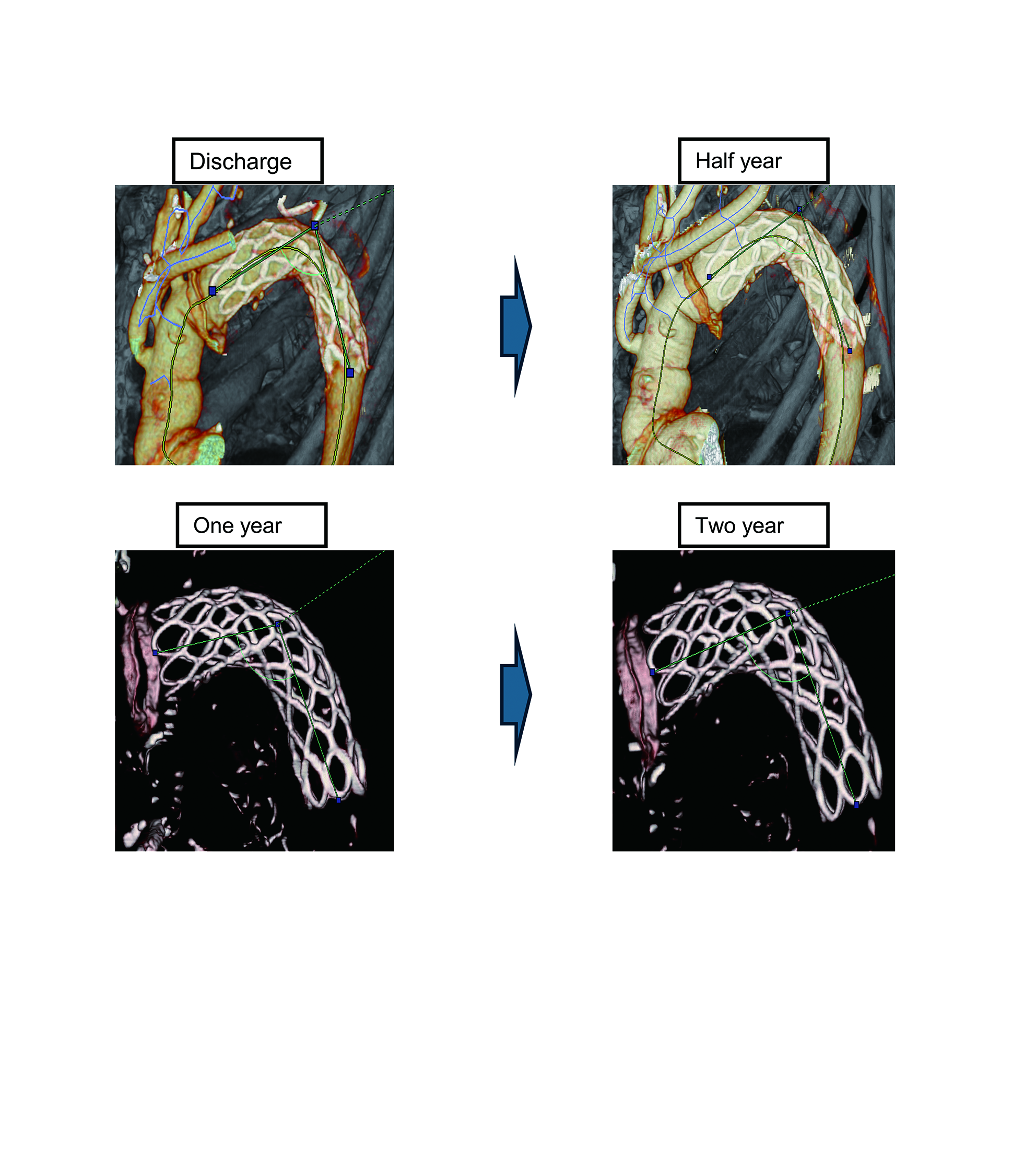

Supplement: ivaf104_Supplementary_Data [file ivaf104_supplementary_data.zip › Supplemental figure4 3rd version.tif]
